# Molecular mimicry revisited: novel perspectives on autoimmune disease initiation and progression

**DOI:** 10.3389/fimmu.2026.1922884

**Published:** 2026-09-22

**Authors:** Urs Christen, Edith Hintermann

**Affiliations:** Institute for Pharmacology and Toxicology, Goethe University Frankfurt, Frankfurt am Main, Germany

**Keywords:** autoimmune disease, epitope, molecular identity, molecular similarity, pathogen infection

## Abstract

Molecular mimicry has long been implicated as one possible mechanism linking pathogen infection to autoimmunity, yet firm proof of its contribution to disease initiation and/or progression remains incompletely defined. In this review, we revisit the concept of molecular mimicry in light of recent advances in immunology, structural biology, and systems medicine. Beyond classical sequence homology, emerging evidence highlights the importance of structural and conformational epitope similarity, post-translational modifications, and context-dependent antigen presentation in shaping cross-reactive immune responses. We discuss how these factors influence the activation of autoreactive T and B cells, the breakdown of immune tolerance, and the establishment of chronic autoimmune disease. We further reevaluate the body of evidence for molecular mimicry as inducer and/or accelerator of specific autoimmune diseases ranging from mere association to proven causative relationship. Thereby, we further examine novel experimental approaches that have refined the identification of mimicry candidates and improved our understanding of their pathogenic relevance. By integrating classical paradigms with contemporary findings, this review provides a detailed outline for understanding molecular mimicry as a dynamic and multi-layered contributor to autoimmune disease.

## Introduction

The first reference of the term “molecular mimicry” appears in a publication from Raymon T. Damian that suggested a sharing of antigenic structures (proteins, lipids, or carbohydrates) by parasite and host as a defense mechanism for pathogens to avoid being recognized by the host’s immune system (1). This concept has been formulated already two years earlier by Rowley and Jenkin who observed an antigenic cross-reactivity between parasite and host and postulated that serum factors, so-called opsonins, might be involved in the pathogenicity (2). As we know now such opsonins comprise a large group of proteins, including complement and antibodies, that tag bacteria or host cells to be recognized by phagocytes (3). Thus, from an evolutionary point of view it is beneficial for a pathogen to share structural similarities with the host to avoid being tagged by opsonins, such as specific antibodies, and to be eliminated eventually. For the host a more aggressive immune response is beneficial, since pathogen elimination is accelerated. Further, an uncompromising immune response is also better in recognizing modified self, such as cancer cells or neo-antigens arising through protein modification by xenobiotics. Thereby a more divers MHC repertoire might guarantee protection from a broader range of pathogens but also increases the probability of cross-reactivity with self-components. Thus, autoimmunity might be the price we pay for a better pathogen defense. The evolution of the immune system is a long-term process with pathogens trying to circumvent the defense mechanisms of the host and the host developing new mechanisms to overcome the new strategies of the pathogens (**Table 1**). Molecular mimicry is just one of many evolutionary adaptations that facilitated pathogen survival. In order to counter this strategy, the host needed to further improve pathogen recognition and elimination, which might have come along with a possible self-reactivity. It seems that for a very long time such a trade-off survival against infection has outweighed later-life autoimmune costs. However, due to the changes in our lifestyle and the environment these costs may get bigger and bigger.

**Table 1:** Co-evolution of pathogen and host.

Phase	Host defense	Pathogen response	Host countermeasures
Preimmune age	• Membranes • Chemical barriers	• Membrane penetration • Horizontal gene transfer	• Selective membrane transport • Early restriction modification
Prokaryotic immunity	• Restriction enzymes • CRISPR-Cas	• Sequence mutations • Anti-CRISPR proteins	• CRISPR diversification • Cas protein variants
Early eukaryotes (Unicellular)	• Phagocytosis • Autophagy	• Intracellular survival (lysosomal resistance) • Hijacking of vesicular trafficking	• Enhancement of lysosomal enzymes • Intracellular pattern recognition
Innate immunity (Multicellular)	• Pattern recognitions receptors (PPR, i.e. TLRs) • Pathogen-associated molecular pattern (PAMP) recognition • Pro-inflammatory factors	• PAMP alteration • PAMP masking • Anti-inflammatory factors	• PPR diversification and redundancy • Recognition of damage- associated molecular patterns (DAMPs)
Innate immunity (Invertebrates)	• Cellular defenses • Antimicrobial peptides (AMP) • RNA interference (RNAi)	• Accelerated replication to exhaust defenses • RNAi suppression	• AMP diversification • RNAi amplification loops
Adaptive immunity and MHC immune surveillance	• B and T cells • VDJ recombination (Antibody diversification) • Antigen presentation (MHC)	• High rate of mutation • Antigenic variation • Phases of latency • MHC downregulation	• Clonal expansion • B and T cell memory • Cytotoxicity • NK cells detect “missing self”
Advanced vertebrate immunity	• Antibody class switching • Mucosal immunity • Somatic hypermutation • Complex cytokine networks	• Molecular mimicry • Exploitation of immune tolerance	• Regulatory T cells • Immune balance
Modern human technology	• Antibiotics • Virostatics • Vaccination	• Antibiotic-Resistance • Virostatic-Resistance • Antigenic escape variants	• New antimicrobial drugs • Strain-matching vaccinations • Multivalent vaccines • Enhancement of T cell immunity

Pathogens have influenced defense mechanisms of the host since the beginning of life. Such a co-evolution of pathogen and host led to the development of the immune system as we know it today. Molecular mimicry turned out to be a selective advantage for pathogens that share similarities of critical epitopes with the host in order to avoid being recognized as foreign. Note that this table is an approximation of the co-evolutionary events based on several reviews on that topic that does not claim to be complete (139–142).

In contrast to the evolution of our immune system, the changes in our environment, as occurring in the last two centuries, are much quicker and leave only very limited time for adaptation. For example, within the lifespan of only a few generations the overall hygiene has changed dramatically for a large portion of humanity from an environment that has constantly exposed our organism to pathogens to clean, almost sterile, conditions in many regions today. In this context the hygiene hypothesis originally stating that allergies occur more frequently in families with only one or two children than in families with many siblings due to their lower exposure to common childhood infections (4) has been expanded to autoimmune diseases in the early 2000s (5). In the meantime, additional factors, such as the microbiome, dysbiosis, and multiple infections, have been integrated into the hygiene hypothesis (6). Thus, in a nutshell: An underemployed immune system may become dysregulated and targets self. Dysbiosis due to a modern lifestyle consisting of low-fiber, highly processed “Western” diet as well as high sugar and fat intake combined with an excessive use of antibiotics and reduced microbiota exposure early in life may contribute to immune system dysregulation (7, 8). Furthermore, chemical pollutants, nanoparticles, and the excessive use of laundry detergents may cause epithelial barrier breaches resulting in microbial translocation and chronic immune activation (9). As demonstrated for *Enterococcus gallinarum*, gut commensial bacteria may themselves cause a barrier breach and translocate to other organs, such as the liver, where they may exacerbate autoimmunity (10). Indeed, *E. gallinarum* has been found in the liver of patients with autoimmune liver diseases and drives autoimmunity in mouse models (11). Thus, molecular mimicry between pathogen and host is just one out of many factors that contribute to the recent dramatic increase in the incidence of autoimmune diseases and allergies. Due to such multifactorial etiology firm proof of a contribution or even a critical role of any of these factors seems difficult. Pathogens may have beneficial (hygiene hypothesis) or detrimental effects on autoimmunity. Here the nature of the pathogens is an important factor. On the one hand, beneficial effects have been largely attributed to parasitic pathogens, including Helminths (12) that elicit a pronounced type 2 immune response and immunoregulatory circuits that largely tolerate the pathogen and thereby reduce tissue damage caused by an overly active immune response (13). On the other hand, intracellular pathogens, such as viruses, that induce a strong type 1 polarity, are with a few exceptions (for example cytomegalovirus seropositivity that is negatively associated with multiple sclerosis (14)) predominantly associated with an initiation or exacerbation of autoimmunity (15). Here, we will review the evidence for molecular mimicry to initiate and/or accelerate certain autoimmune diseases. In addition, we would like to display how our current understanding of the molecular mimicry concept has changed from the initial postulation in the early 1960s and the first identification of sequence similarities in the 1980s.

## Pathogens as drivers of autoimmunity

Pathogens have been widely associated with virtually all autoimmune diseases in the past. However, firm proof for pathogens as actual drivers of autoimmunity is rather difficult due to the following reasons: First, not all infected individuals develop the corresponding autoimmune disease or autoimmunity remains asymptomatic. Second, pathogens might act as “hit-and-run” events leaving no traces of their prior presence by the time of disease diagnosis. Third, every individual encounters numerous infections within their lifetime, some of which might leave traces of their existence or are even persistently present, but have no impact on autoimmunity. Fourth, epidemiologic observations and experimental evidence suggest that some pathogens have the potential to prevent or abrogate autoimmune processes. This circumstance is also supported by the hygiene hypothesis. Last, it is likely that for many autoimmune diseases not a single but multiple infectious events might be involved in their etiology. Considering that the burden of proof for pathogens to drive autoimmunity is stringent and often relies on information that is difficult to gather, it seems clear that demonstrating a critical role of molecular mimicry as a mechanism by which such pathogens indeed brake self-tolerance is even more problematic. At the end, molecular mimicry is just one of many possible mechanisms by which pathogens might be involved in the development of autoimmune diseases. For example, pathogen infections can cause the release of previously sequestered antigens or uncovering of cryptic epitopes to which no central tolerance has been established. Bystander activation through pro-inflammatory cascades may activate anergic, self-reactive T cells polyclonally. Similarly, bacterial superantigens may cross-link MHC and T cell receptor (TCR) without the requirement of a presented peptide. Further, pathogens might cause bystander suppression of regulatory T cells resulting in a shift of the immune balance towards a more aggressive response (16).

## Mechanism of molecular mimicry

As such molecular mimicry appears to be a widespread phenomenon. Forty years ago, Sirinivasappa et al. screened more than 600 monoclonal antibodies raised against 11 different viruses and found that 3.5% of the antibodies reacted to specific cell types in organs from normal, uninfected mice (17). However, this study only demonstrated that the cross-reactivity of these anti-virus antibodies was sufficient to elicit an immunofluorescent signal on mouse tissue sections that has been considered “positive” or “weakly positive” (17). For molecular mimicry to indeed induce and/or accelerate autoimmunity several requirements must be fulfilled: First, the host has to be exposed to environmental factors, such as pathogens or xenobiotics. Second, such environmental factors need to share a structural similarity to one or more host components. Third, antibodies and/or T cells should exhibit cross-reactivity within a critical range of affinity. In this context, it is important to acknowledge that due to the high level of B cell receptor (BCR) and TCR degeneracy cross-reactivity may also occur for peptide sequences with only moderate similarity (18). In pathogen infections, sequential or conformational similarities between host and pathogen proteins are most frequently observed. In contrast, similar to hapten-carrier complexes known from hypersensitivity reactions, xenobiotics would need to modify self-proteins to form protein-adducts that share similarities to endogenous self-protein modifications. However, the mere formation of neo-antigens by chemicals does not necessarily result in molecular mimicry of (other) self-components. The formation of neo-antigens is well-documented for chemicals, such as the antihypertensive hydralazine, the antiarrhythmic agent procainamide, or the anesthetic agent halothane. Reactive metabolites of hydralazine and procainamide form neo-antigens that may cause drug-induced lupus erythematosus (19). Similarly, halothane, when metabolized by cytochrome P450 2E1 generates trifluoroacetylated-proteins (TFA-proteins) that are recognized as neo-antigens by the immune system and may cause an autoimmune-mediated halothane hepatitis in a minority of individuals (20). Initially, the immune response is directed against the protein modification itself; however, owing to epitope spreading, subsequent reactivity to unmodified epitopes results in chronic autoreactivity against the native proteins (21). Interestingly, TFA-proteins have endogenous human counterparts that can serve as molecular mimicry targets, namely enzymes of the 2-oxoacide dehydrogenase family, including the E2-subunit of the pyruvate dehydrogenase complex (PDC-E2) (22, 23). In this case, patients’ cross-reactive antibodies recognize the lipoic acid prosthetic group of PDC-E2 (24). Interestingly, patients with halothane hepatitis were found to exhibit aberrant expression of the cross-reactive self-component PDC-E2 (25). Thus, in the majority of individuals molecular mimicry seems to instigate protection, rather than damage, by establishing immune tolerance not only to PDC-E2, but also to its mimicry counterpart, the TFA-modification.

A well-characterized case of pathogen-induced protein modification is the citrullination of proteins by the oral pathogen *Porphyromonas gingivalis* through the bacterial peptidylarginine deiminase (26). The resulting citrullinated bacterial proteins resemble citrullinated host proteins, such as citrullinated α-enolase, and may contribute to the progression of rheumatoid arthritis (RA) through the generation of anti-citrullinated protein antibodies (ACPA) (27). Other post-translational modifications, including carbamylation, acetylation, or transglutaminase-mediated deamidation may also be important in generating neo-epitopes to which cross-reactive antibodies bind or which may alter peptide-MHC binding. Under inflammatory conditions carbamylation of proteins results in the conversion of lysine residues to homocitrullin. In RA the presence of homocitrullin may cause the formation of anti-carbamylated protein (anti-CarP) antibodies that are, like ACPA, cross-reacting with citrullinated α-enolase (28, 29). Further, acetylation of lysine-residues to form acetyl-lysine may result in the generation of anti-acetylated protein antibodies (AAPA). In RA-patients all these anti-modified protein antibodies (AMPA) show considerable cross-reactivity with citrullinated, homocitrullinated/carbamylated, and acetylated proteins (30).

Another interesting post-translational modification that may result in molecular mimicry is transglutaminase 2 (TG2)-mediated deamidation of glutamine to glutamate, which may occur within gluten peptides. Deamidated gluten peptides bind particularly well to HLA-DQ2 and HLA-DQ8, the major genetic susceptibility molecules for celiac disease (CD) (31). Thus, TG2-mediated gluten modification increases its immunogenicity and may even result in the generation of anti-TG2 autoantibodies, possibly through initial recognition of TG2-gluten complexes (32, 33). Anti-TG2 autoantibodies are the hallmark autoantibodies in CD and plasma cells secreting TG2-specific IgA autoantibodies have been found in high abundance in CD intestinal lesions (34).

As for deamidated gluten peptides, the MHC haplotype has a tremendous impact on molecular mimicry. Cross-reactivity can only occur if both mimicry partners are properly presented within the available MHC molecules. Indeed, rheumatic fever following group A streptococcal infection is associated with the presence of HLA-DR4, HLA-DR7, and HLA-DQ alleles (see below for more details on the evidence of molecular mimicry in rheumatic fever) (35). MHC haplotype susceptibility is more important for cross-reactive T cells but may also influence cross-reactive B cells through its effect on T cell help. Historically, because patients’ serum samples were relatively easy to analyze, more putative cases of molecular mimicry based on sequence homology were identified and verified for cross-reactive antibodies than T cells. However, only a minority of autoimmune diseases, such as Guillain–Barré Syndrome (GBS) or acute rheumatic fever (ARF), are predominantly autoantibody-mediated. Interestingly, GBS and ARF are diseases for which the strongest evidence exists for molecular mimicry as a driver of the disease (see below). In contrast, the majority of autoimmune diseases, including type 1 diabetes, multiple sclerosis, or autoimmune uveitis, are predominantly propelled by tissue-destructive autoreactive T cells. Indeed, detailed assessment of patients’ T cell repertoires confirmed the presence of cross-reactive T cells that target predicted epitopes of both pathogen and host. Thus, autoreactive T cells seem to be central orchestrators of molecular mimicry-driven autoimmunity (36).

Overall, the degree of similarity of linear epitopes seems critical. Too low a similarity would result in a low affinity recognition by cross-reactive T cells. In contrast, a very high similarity, or even identity, might prevent T cell recognition due to a high degree of tolerance originating from the negative selection in the thymus. The “sweet spot” for molecular mimicry might therefore lie in a region with sufficiently high similarity, but without complete identity (**Figure 1**). Lateral mechanisms like epitope spreading or bystander activation may further alter the location of such a sweet spot, by changing the epitope range or providing additional activation factors, respectively. Indeed, in the CYP2D6 model for autoimmune hepatitis (AIH), in which an infection of wild-type mice with an adenovirus encoding for the human liver autoantigen cytochrome P450 2D6 (CYP2D6) breaks tolerance to murine Cyp homologues (37), the T cell reactivity is greatest toward epitopes with a sequence homology of 50-70% (see in detail below) (38).

**Figure 1:**
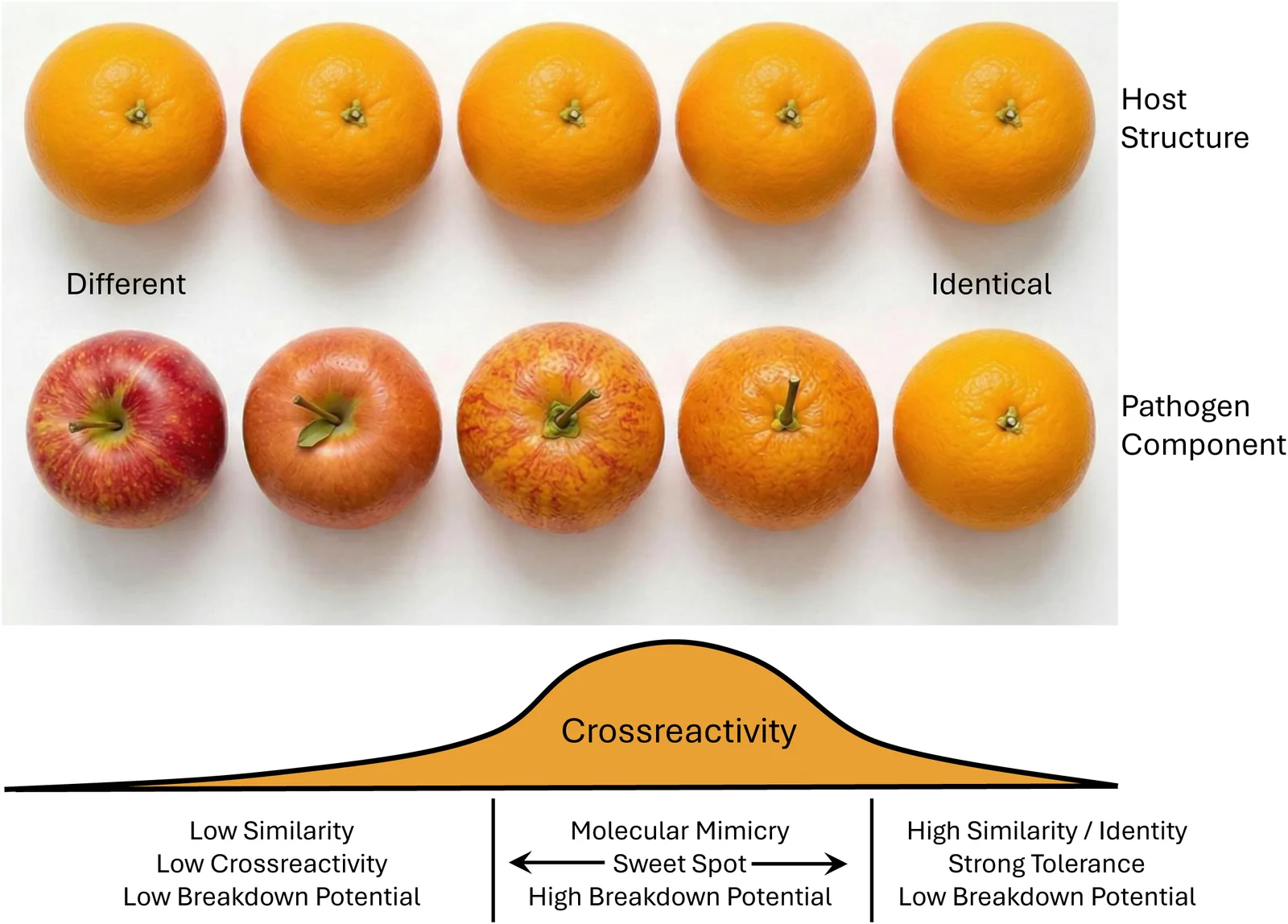
Sweet spot of molecular mimicry by T cell epitopes. Pathogens with components that share similarities with structures of the host may break immune self-tolerance of the host. Whereas B cell epitopes are often conformational and may be of miscellaneous origin (proteins, carbohydrates, DNA, etc.), T cell epitopes mostly consist of short linear peptides. Due to the degenerate nature of the TcR many similar peptides embedded in the MHC peptide binding groove can be recognized and result in T cell activation. Thereby the degree of similarity seems crucial: Too low a similarity is insufficient due to low cross-reactivity; too high a similarity is counteracted by a strong tolerance. Molecular mimicry provides a similarity sweet spot that is sufficiently cross-reactive but does not have to overcome a strong tolerance. Mechanisms such as epitope spreading and bystander activation might shift this hot spot. (The apple to orange conversion has been generated using deevid.ai.).

## Proven and proposed cases of molecular mimicry

Already in 1957, Witebsky et al. postulated criteria for autoimmune diseases that included autoantibodies and/or cell-mediated autoimmunity, identification of related autoantigens, as well as experimental reproduction of the autoimmune response and the corresponding pathological changes (39). More than 30 years later these criteria have been revised by Noel R. Rose and Constantin Bona considering all the new knowledge gained from molecular biology and hybridoma technology (40). They adapted the original Witebsky criteria into three categories of evidence for an autoimmune etiology: i) Direct evidence from transfer of pathogenic antibody or pathogenic T cells; ii) indirect evidence based on reproduction of the autoimmune disease in experimental animals; iii) circumstantial evidence from clinical clues, such as association with other autoimmune diseases, lymphocytic infiltration of target organs, association with a particular MHC haplotype, or favorable response to immunosuppression (40). With regards to molecular mimicry and autoimmunity it gets more complex, but there is a general agreement for proof of molecular mimicry as an inducer and/or accelerator of autoimmunity that has been reviewed in many articles (see for example (36)). In a simplified view, the following four conditions should be fulfilled: First, the existence of linear or conformational similarities between epitopes of the host and the environmental trigger (i.e. pathogen or xenobiotic). Second, the detection of cross-reactive antibodies and/or T cells that recognize both mimicry counterparts. Third, an epidemiologic link between exposure to the environmental trigger and the occurrence of autoimmunity. Fourth, reproducibility of autoimmunity in a model system, including the exposure of experimental animals to the environmental trigger. As described above, the burden of proof for molecular mimicry to be a major pathogenic driving force is high. However, there are several autoimmune-mediated diseases for which sufficient evidence has been collected to imply a decisive role for molecular mimicry as precipitating mechanism for the disease (see also **Table 2**).

**Table 2:** Most prominent examples for molecular mimicry as drivers of autoimmunity.

Autoimmmune disease	Pathogen / trigger	Pathogen Component	Host tissue	Host component	Evidence	References
Guillain-Barré Syndrome	*Campylobacter jejuni*	Lipooligo-saccharides (LOS)	Peripheral nervous system	Peripheral nerve gangliosides (GM1)	Epitope similarity: Fulfilled Demonstrated cross-reactivity: Fulfilled (Ab) Epidemiologic association: Fulfilled Reproduction in experimental model: Fulfilled *Additional evidence*: Antibody transfer induces disease	(41–46)
Rheumatic Fever	*Streptococcus pyogenes*	M-protein	Heart	Cardiac myosin	Epitope similarity: Fulfilled Demonstrated cross-reactivity: Fulfilled (T cells & Ab) Epidemiologic association: Fulfilled Reproduction in experimental model: Fulfilled	(35, 47–51)
Multiple Sclerosis	Epstein-Barr Virus	EBNA-1	CNS	CRYAB GlialCAM	Epitope similarity: Fulfilled Demonstrated cross-reactivity: Partially fulfilled (T cells & Ab) Epidemiologic association: Fulfilled (very strong) Reproduction in experimental model: Partially fulfilled (antigen immunization only)	(52–58)
Systemic Lupus Erythematosus	Epstein-Barr Virus Commensal bacteria	EBNA-1 Bacterial Ro60	Systemic	Human Ro60	Epitope similarity: Fulfilled Demonstrated cross-reactivity: Fulfilled (T cells & Ab) Epidemiologic association: Partially fulfilled Reproduction in experimental model: Fulfilled	(59–62)
Type 1 Diabetes	Enterovirus, Coxsackie virus B4, Cytomegalovirus, Rotavirus	VP1, VP7, MDBP, p2C	Pancreas	GAD65 IA-2	Epitope similarity: Fulfilled Demonstrated cross-reactivity: Partially fulfilled (controversial) Epidemiologic association: Fulfilled Reproduction in experimental model: Partially fulfilled (model antigens only)	(63–73)
Rheumatoid Arthritis	*Porphyromonas gingivalis*	Enolase	Joints	Human α-enolase	Epitope similarity: Fulfilled Demonstrated cross-reactivity: Fulfilled (Ab) Epidemiologic association: Fulfilled Reproduction in animal model: Fulfilled	(74–78)
Autoimmune Hepatitis	Hepatitis C Virus, Epstein-Barr Virus, Cytomegalovirus, Herpes simplex virus 1	HCV core, E2, NS3, NS5a	Liver	Cytochrome P450 2D6	Epitope similarity: Fulfilled Demonstrated cross-reactivity: Fulfilled (Ab) Epidemiologic link: Partially fulfilled for some viruses Reproduction in experimental model: Partially fulfilled (model viruses encoding human autoantigens)	(37, 80, 84–91)
Primary Biliary Cholangitis	*Novospingobium* *aromaticivorans*, *Escherichia coli*, Food additive	46- and 50kDa proteins, bacterial PDC-E2, 2-octoynoic acid	Liver	Pyruvate dehydrogenase complex E2 (PDC-E2), Lipoic acid	Epitope similarity; Fulfilled Demonstrated cross-reactivity: Fulfilled (T cells & Ab) Epidemiologic link: Partially fulfilled (indirect evidence) Reproduction in animal models: Fulfilled only for 2-octoynoic acid	(92–99)
Behçet's disease	*Streptococcus sanguinis*	HSP65	Skin, Eye, Mouth, Genitals	HSP60	Epitope similarity: Fulfilled Demonstrated cross-reactivity: Fulfilled (T cells & Ab) Epidemiologic link: Fulfilled Reproduction in animal models: Partially fulfilled	(100–106)

This table lists the evidence for molecular mimicry to play a crucial role in the pathogenesis of a few selected examples of autoimmune diseases. Note that this is only a selection of reported cases and that some diseases (e.g. multiple sclerosis) have been associated with other pathogen infections as well.

### Guillain–Barré syndrome

The clearest example for molecular mimicry to cause autoimmunity is the development of Guillain–Barré Syndrome (GBS) after infection with *Campylobacter jejuni* (41, 42). First, there is structural proof that *C. jejuni* lipooligosaccharides (LOS) mimic peripheral nerve GM1 ganglioside (43). Second, patients generate cross-reactive antibodies recognizing LOS as well as the GM1 ganglioside upon *C. jejuni* infection (44). In this context, a correlation of specific antibody profiles with different bacterial strains has been detected (44). Third, passive transfer of patient antibodies in animal models and immunization of rabbits with purified gangliosides or *C. jejuni* lipo-oligosaccharide induced neurological disorder resembling human GBS (45). In summary, *C. jejuni*-induced GBS may be considered as a *bona fide* example of molecular mimicry in humans. Interestingly, in GBS molecular mimicry is restricted to cross-reactive B cells/antibodies reacting to nearly-identical carbohydrates and does not require cross-reactive T cells to precipitate the disease. However, it has been recently demonstrated that autoreactive T cells may very well contribute to the GBS variant with a demyelinating phenotype (46). Indeed, such autoreactive T cells target myelin antigens of the peripheral nerves and have been detected in the nerve biopsy from a patient with GBS (46).

### Acute rheumatic fever

Also, strong evidence, though slightly less structurally precise than GBS, exists for acute rheumatic fever (ARF) caused by an infection with *Streptococcus pyogenes* (35). The epidemiologic link between ARF and *S. pyogenes* is very consistent and has been reported globally (47, 48). Here the *S. pyogenes* M protein shares epitopes with cardiac myosin and cross-reactive antibodies and T cells have been identified in patients (49). Such cross-reactive antibodies indeed bind to heart valve tissue (49). In addition, N-acetyl-β-D-glucosamine, the dominant epitope of the group A streptococcal carbohydrate, confers molecular mimicry to lysoganglioside of the host and patients with Sydenham chorea, a major neurological manifestation of ARF, generate cross-reactive antibodies reacting to both N-acetyl-β-D-glucosamine and lysoganglioside (50). Importantly, ARF-like disease has been induced by immunization with group A streptococcus in rat models (51).

### Multiple sclerosis

Multiple sclerosis (MS) was one of the first autoimmune diseases for which molecular mimicry has been suggested as a disease initiating mechanism. In particular, Robert Fujinami and Michael Oldstone demonstrated the existence of a sequence similarity between hepatitis B virus (HBV) polymerase and myelin basic protein (MBP), one of the major autoantigens recognized by antibodies and T cells of patients with MS (52). Experimentally, this observation has been verified in rabbits that were injected with HBV polymerase peptide. The rabbits generated cross-reactive antibodies to HBV polymerase and MBP and isolated peripheral blood mononuclear cells (PBMC) proliferated in response to both HBV polymerase and MBP. In addition, a histological examination of CNS tissue revealed an experimental allergic encephalomyelitis (EAE)-like disease pattern (52). However, it has since not been demonstrated that natural HBV-infection can cause MS. Recent evidence supports the suggested general molecular mimicry concept in MS, but the focus changed from the initial HBV-polymerase – MBP mimicry pairing to Epstein-Barr Virus (EBV).

Due to its high prevalence EBV has been associated with many autoimmune diseases. Recently, additional evidence has been collected for EBV-induced MS. Here the burden of proof is particularly high, since molecular mimicry is clearly just one out of many factors influencing MS pathogenesis and besides EBV, infection with other pathogens, including HHV-6, have been associated with MS (53). In context with EBV, the Epstein–Barr virus nuclear antigen 1 (EBNA-1) shares sequence homology with CNS proteins, such as alpha-crystallin B (CRYAB) and glial cell adhesion molecule (GlialCAM). MS patients generate cross-reactive antibodies and T cells recognizing EBNA-1 as well as CRYAB and GlialCAM (54, 55). Interestingly, a higher EBNA-1 antibody titer correlates with an increased relapsing-remitting MS activity (56). A recent study in 30 individuals with MS and 30 matched controls suggests that the antibody response to EBNA-1 may even be the highest serologic risk factor for MS (57). The strongest evidence for an involvement of EBV-infection in the pathogenesis of MS comes from a huge longitudinal study involving >10 million young adults of a U.S. military cohort that have been monitored over a 20-year period (58). The big advantage of this study was that it included 5.3% (corresponding to >500’000 individuals) that were EBV-negative at the time of the first serum sample. The study demonstrated that the risk of MS increased 32-fold after infection with EBV, but was not increased after infection with other viruses, including the similarly transmitted cytomegalovirus. Importantly, serum levels of neurofilament light chain, a biomarker of neuroaxonal degeneration, increased only after EBV seroconversion (58). Overall, EBV is currently the strongest single environmental risk factor for MS and may best be interpreted as a necessary trigger within a multifactorial model, requiring MHC susceptibility, environmental co-factors, and immune regulation impairment.

### Systemic lupus erythematosus

EBV has also been linked to systemic lupus erythematosus (SLE) due to a serological association of the presence of anti-EBV-viral capsid antigen (VCA), presence of EBV DNA, and cross-reactivity of EBV proteins with SLE-associated autoantigens (59). In particular, the EBNA-1 contains several regions with similarities to the human Ro60 antigen, which is a ring-shaped RNA binding protein that forms ribonucleoprotein (RNP) complexes with Y RNAs (60). Anti-Ro60 antibodies are the earliest and most common preclinical anti-nuclear antibodies in patients with SLE and have been demonstrated to be pathogenic (61, 62). Interestingly, Ro60 is highly conserved in evolution and human Ro60 orthologs can be found in multiple commensal bacteria identified on the skin, oral mucosa, and gut including species of *Corynebacterium, Propionibacterium*, and *Bacteroides*. Indeed, patients with SLE carry anti-human Ro60 antibodies as well as T cells cross-reactive with commensal Ro60 orthologs (60). Most compellingly, when germ-free mice were monocolonized by a *Becteroides* species, they generated anti-human Ro60 antibodies and developed signs of SLE-like disease (60). These findings indicate that not only external triggers, such as EBV-infection, but also dysbiosis that favors some commensal species over others might be involved in molecular mimicry-driven autoimmunity.

### Type 1 diabetes

The role of pathogen infection in type 1 diabetes (T1D) is still a matter of debate, but there is growing evidence that viruses, such as enteroviruses (EV) or rotaviruses (RV) indeed play a critical role in T1D (63–65). Debates have been fueled by contradictory findings. For example, a strong association has been found between RV infection in young infants and the first occurrence of islet autoantibodies (65). However, in another study no difference in diabetes-associated autoantibody generation was found in susceptible children who either had or had not been infected with RV (66). For EV, particularly Coxsackieviruses, have been epidemiologically linked to T1D for a long time. Two recent meta-analyses revealed that EV-infection is indeed significantly associated with islet autoimmunity and the development of T1D (67, 68). Importantly, with regards to molecular mimicry, sequence homologies with human T1D autoantigens, such as glutamic acid decarboxylase 65 (GAD65) and the tyrosine phosphatase insulinoma-associated antigen 2 (IA-2), exist in viral proteins like the viral protein 1 (VP1) of EV (69), the protein 2C (p2C) of coxsackie virus B4 (CVB4) (70), the major DNA-binding protein (MDBP) in human cytomegalovirus (HCMV) (71), and the viral protein 7 (VP7) of RV (72). Unfortunately, GAD65-specific T cells isolated from recent onset T1D patients did not cross-react to the homologous peptide of p2C of CVB4 (73). Nevertheless, CVB4 is the leading candidate for a critical contribution of molecular mimicry to the pathogenesis of T1D. However, it seems clear that in contrast to GBS, many other mechanisms, such as persistent viral infection, β-cell stress, bystander activation, and epitope spreading may also be required to further drive the disease process (63).

### Rheumatoid arthritis

Many pathogens including bacteria such as *Porphyromonas gingivalis* (*P. gingivalis*), *Proteus mirabilis* (*P. mirabilis*), and *Escherichia coli* (*E. coli*), as well as viruses, such as EBV, HBV, hepatitis C virus (HCV), and human immunodeficiency virus (HIV), have been associated with RA (74). Interestingly, the strongest association has been shown for *P. gingivalis* that is a major cause for periodontitis (75). Molecular mimicry (~82% sequence similarity) has been detected between *P. gingivalis* enolase and human α-enolase and indeed cross-reactive antibodies have been detected in patients with RA (76). Importantly, direct experimental infection of mice or rats with *P. gingivalis* or immunization of mice with citrullinated or un-citrullinated *P. gingivalis* enolase or human α-enolase breaks tolerance to mouse α-enolase and causes RA-like disease (77, 78).

### Autoimmune liver diseases

Two very interesting cases of molecular mimicry are discussed for autoimmune liver diseases (ALD). There are four well-defined ALD, namely autoimmune hepatitis (AIH), primary sclerosing cholangitis (PSC), primary biliary cholangitis (PBC), and immunoglobulin G4 (IgG4)-associated cholangitis (IAC) that are characterized by a progressive destruction of the liver parenchyma and/or the hepatic bile ducts and the development of chronic fibrosis (79). Patients with type 1 AIH generate anti-nuclear antibodies (ANA) recognizing a broad spectrum of nuclear targets, including DNA, nucleosomes, and histones (80). Similarly, many patients with PSC generate perinuclear anti-neutrophil cytoplasmic antibodies (pANCA) for which a diffuse pattern of target autoantigens, including myeloperoxidase (MPO), proteinase-3 (PR3), lactoferrin, high-mobility group proteins (HMG1/HMG2), and β-tubulin isotype 5 (TBB5), have been suggested, but their actual involvement in the pathogenesis remains unclear (81, 82). For IAC several candidate target antigens for the generated IgG4 antibodies have been reported, but there is no evidence for characteristic pathogenic autoantibodies (83). In contrast, patients with PBC and type 2 AIH recognize clearly defined autoantigens, including PDC-E2 and CYP2D6 as well as formiminotransferase cyclodeaminase (FTCD), respectively (80).

The first case of molecular mimicry has been reported for type 2 AIH, for which many virus infections, including hepatotropic viruses such as hepatitis viruses, EBV, and CMV have been reported to increase the disease risk and are epidemiologically linked to the disease onset (84–87). Molecular mimicry has been detected on the level of cross-reactive type 1 liver-kidney microsomal antibodies (LKM1) recognizing CYP2D6 that have been identified in patients with HCV infection (80, 84). The immunodominant CYP2D6 epitope was indeed recognized by sera of both patients with AIH type 2 as well as patients with an HCV infection (88). Further, cross-reactivities of LKM1 have been demonstrated for herpes simplex virus 1 (HSV-1) and CMV. In HCV, the core, E2, NS3, and NS5a proteins share sequence homology with CYP2D6 and patients’ LKM1 cross-reacted with NS3 and NS5a in immunoprecipitation experiments (84). Molecular mimicry of human cytochrome P450 by HCV has been also reported, however, only for CYP2A6/2A7, but not for the actual human autoantigen CYP2D6 (89). A direct reproduction of HCV, HSV-1, EBV, or CMV-triggered AIH-like disease in an animal model is still missing, but many models used infections with viruses encoding human liver autoantigens to break tolerance and induce AIH-like disease (see below) (37, 90, 91).

The second ALD for which molecular mimicry has been implicated in its etiology is PBC. In patients with PBC infections mostly with bacteria such as *E. coli, Novosphingobium aromaticivorans (N. aromaticivorans)*, and *Lactobacillus delbrueckii*, but also with viruses like HIV, have been associated with the disease (92). For *E. coli* PDC-E2 it has been found that it shares considerable sequence homology with the immunodominant region of human PDC-E2 (93). However, PDC-E2 is a very essential enzyme that shuffles pyruvate into the citrate-cycle and such ubiquitous enzymes often contain extensive sequence homologies between species. Interestingly, the conserved and immunodominant region of PDC-E2 is located around the central lysine residue that carries lipoic acid as prosthetic group, catalyzing the dehydrogenation of pyruvate (94). Nonetheless, anti-mitochondrial antibodies (AMA) generated by PBC patients cross-react with both the human and *E.coli* PDC-E2 versions (93). In addition, T cell clones have been isolated from patients with PBC that cross-react with human and *E. coli* PDC-E2 (95). An epidemiologic association has been reported not with *E.coli* itself, but with urinary tract infection (UTI), in which *E.coli* is the dominant pathogen (96, 97). Due to a lack of further *in vivo* experiments, it remains unclear if *E. coli* PDC-E2 indeed plays a critical role in the etiology of PBC or if the observed tolerance breakdown to human PDC-E2 and the AMA cross-reactivity is just the result of another pathogens infection. Indeed, two proteins of *N. aromaticivorans* also exhibit molecular mimicry with human PDC-E2. These proteins have been referred to as 46-kDa or 50-kDa protein and their identity has only partially been revealed as lipoylated PDC-E2-like proteins (92). Interestingly, sera from AMA-positive PBC patients showed a reactivity to *N. aromaticivorans* that was 100-1000-fold higher than to *E.coli* (98). Furthermore, a chemical xenobiotic, the cosmetic and food additive 2-octoynoic acid (2-OA) shows a high structural similarity to lipoic acid as present in PDC-E2. AMA of PBC-patients cross-reactively recognized both lipoylated as well as 2-OA-modified proteins. Indeed, injection of 2-OA coupled to bovine serum albumin (BSA) into wild-type C57BL/6 mice resulted in the generation of PBC-like disease characterized by autoimmune cholangitis, typical AMA, elevated serum TNFα and IFNγ, and liver infiltration by activated CD8 T cells (99).

### Behçet’s disease

Behcet’s disease (BD) is a systemic inflammatory disease that affects multiple locations and is characterized by recurrent symptoms of oral aphthosis, genital ulcers, skin lesions, and uveitis (100). BD has been associated with microbial infection, in particular with *Streptococcus sanguinis* (*S. sanguinis*) that has been isolated from patients with BD (101). This epidemiologic association has been confirmed by modern microbiome analyses that focused on oral microbiota dysbiosis (reviewed in (102)). Molecular mimicry has been identified between *S. sanguinis* heat shock protein 65 (HSP65) and its human counterpart heat shock protein 60 (HSP60) which was found to be overexpressed in skin lesions (103). Patients’ antibodies cross-react with both HSP65 and HSP60 and in infected individuals the T cell response to HSP65 is stronger in BD patients than in healthy controls (100). Furthermore, challenge of Lewis rats with HSP65 peptide caused mild to moderate uveitis-like symptoms, but clinical involvement of the mouth, skin, external genitalia, joints, or the neurological system was not observed (104). Interestingly, *S. sanguinis* also induces NETosis by neutrophils, a pro-inflammatory defense mechanism against bacteria that has been described to be triggered in excess in BD patients (102). *S. sanguinis* has a particularly strong effect on neutrophil activation via SpxB pyruvate oxidase-mediated bacterial H_2_O_2_ production that causes an enhanced production of reactive oxygen species (ROS) and NETosis in neutrophils (105). Importantly, neutrophil extracellular traps (NET) contain besides DNA and histones other components like antimicrobial peptides (including α-defensins and LL-37/cathelicidin), MPO and neutrophil elastase (NE), which potentiate the local inflammatory milieu (106). Thus, as for many other autoimmune diseases molecular mimicry might not be the only mechanism that is involved in the immunopathology of BD.

Besides this small selection of well-characterized molecular mimicry cases, many other autoimmune diseases have been associated with molecular mimicry, including Sjögren’s syndrome (107), psoriasis (108), systemic sclerosis (109), autoimmune thyroiditis (110), myasthenia gravis (111), stromal keratitis (112), and autoimmune uveitis (113). However, in contrast to *C. jejuni*-induced GBS or *S. pyogenes*-induced ARF, firm proof for an actual mechanistic role in precipitating autoimmunity is limited and therefore still a matter of debate. The mere existence of sequence homologies between proteins of pathogen and host, as reported in hundreds of articles, is not sufficient.

## New insight from the COVID-19 pandemic

The COVID-19 pandemic can be considered as a natural experiment at global scale that provided massive, real-time datasets and dramatically accelerated research into molecular mimicry. Longitudinal analyses enabled the detection of *de novo* autoantibody formation targeting diverse self-proteins even in mild cases (114) and an increased incidence of autoimmune diseases (115). Severe acute respiratory syndrome coronavirus 2 (SARS-CoV-2) caused an elevation of various autoantibodies that target specific autoantigens in many infected individuals (116, 117). Molecular mimicry has been predominantly identified on the level of epitope similarity between the spike protein and the replicase polyproteins 1a and 1ab of SARS-CoV-2 and several human proteins (115). Especially for the spike protein many sequence homologies to a broad diversity of targets, including dishevelled-associated activator of morphogenesis 2 (DAAM2), neural cell adhesion molecule L1-like protein (CHL1), T cell immunoglobulin and mucin-domain 3 (HAVR2/TIM3), follistatin related protein 1 (FSTL1), FH1/FH2 domain-containing protein 3 (FHOD3), unconventional myosin-XVIIIa (MYO18A), elastin microfibril interfacer 3 (EMILIN3), lysosome associated membrane protein 1 (LAMP1), and epithelial sodium channel (αENaC), have been predicted to be recognized by B cell receptors (118). In the same study, several HLA class II genetic variants were computationally associated with a higher likelihood of cross-reactive immune reactions following COVID-19, and seven T helper cell autoepitopes were identified that could be implicated in autoimmune T cell responses through presentation by the associated class II HLA molecules (118).

However, though very thought-provoking such *in silico* findings need to be backed up with actual experimental data. The COVID-19-era research on molecular mimicry shows that large datasets can be processed and computed very quickly, but it also exposed that the same kind of limitations than forty to fifty years ago still exist. As a result, many studies merely report sequence similarities and do not perform functional validation, which paves the way for overinterpretation as cross-reactivity, epidemiologic association, or reproduction in animal models are often missing. This problem has been further analyzed in two recent reviews by Adiguzel et al. and Arevalo-Cortez et al. who sorted out those studies reporting molecular mimicking that have been experimentally validated, and immunological cross-reactivity may indeed account for autoimmunity development in these cases (114, 119). Both reviews came to the conclusion that, with very few exceptions, most published studies on the generation of autoantibodies after SARS-CoV-2 infection in the context of molecular mimicry are based on sequence homologies and *in silico* simulations. In connection, possible human target autoantigens have been identified that might be involved in autoimmune diseases, such as alopecia areata, ankylosing spondylitis, autoimmune thrombocytopenic purpura, autoimmune uveitis, Graves’ disease, MS, and T1D (115). Although there are experimental studies that demonstrated cross-reactive antibodies and T cells (119) comprehensive studies linking sequence homologies, bioinformatic prediction, epidemiologic association, experimental evidence for cross-reactivity, and reproduction of the findings in animal models are still missing. The COVID-19 pandemic started 7 years ago and already by September 2021 an estimated global SARS-CoV-2 seroprevalence from infection or vaccination of 59.2% was reached (120). Thus, considering that environmental triggers that participate in the breakdown of self-tolerance and the development of autoimmunity might occur decades before the clinical manifestation of the disease there might be more solid evidence for SARS-CoV-2 infection as a driver in the future. It will also be interesting to see how much molecular mimicry is involved in the long COVID syndrome that is likely to arise from a combination of overlapping mechanisms, including persistent viral reservoirs or antigens, immune dysregulation and chronic inflammation, reactivation of latent viruses, microvascular dysfunction and clotting abnormalities, autonomic nervous system dysfunction, and autoimmunity, potentially including molecular mimicry (121). It has been recently demonstrated that 60% of new-onset autoantibodies remained elevated for at least 12 months after SARS-CoV-2 infection (122). Interestingly, these autoantibodies showed a large diversity and interindividual heterogeneity targeting a wide range of antigens across the proteome. In addition, the main epitopes display sequence similarities indicative of molecular mimicry with the highly conserved fusion peptide of the SARS-CoV-2 spike protein (122). An autoimmune, molecular mimicry-related component in Long COVID syndrome would require such a persistent presence of cross-reactive antibodies and/or T cells unless extensive epitope spreading causes a loss of SARS-CoV-2-specificity over time. Whereas, T cells largely depend on linear epitopes, antibodies reacting with conformational, rather than linear, epitopes might be more persistent in the context of molecular mimicry. A possible human mimicry counterpart of, for example, the SARS-CoV-2 spike protein remains accessible for B cells long after the virus is eliminated. One could speculate that thereby the chances for antibodies recognizing conformational epitopes seems higher since they are not dependent on the presence of a narrow spectrum of conserved amino acid sequences. Intravenous immunoglobulin (IVIG) therapy has been used to treat COVID-19-associated autoimmune and inflammatory diseases with variable but often favorable results (123) and even patients with Long COVID syndrome with persistent immune-mediated symptoms showed promising clinical improvement (124). One of the major effects of IVIG therapy is the blockade of Fc-receptors and thereby preventing antibody recycling. Indeed, IVIG therapy has been demonstrated to enhance the clearance of autoantibodies and to reduce their *in vivo* persistence (125).

In general, molecular mimicry gained much more attention following the COVID-19 pandemic than before, catalyzing an acceleration of predictive autoimmunity through bioinformatic analysis of protein homology. Integration of genetic susceptibility using precise HLA-typing and machine learning to rank “high-risk” molecular mimicry epitopes allow *in silico* simulation of immune responses and the identification of pathogen peptides most likely to trigger autoimmunity in susceptible individuals. However, one has to be aware that many other factors, such as immune dysregulation,

bystander activation, cytokine storm, epitope spreading, host genetic predisposition, and viral genomic variability are potentially involved in linking COVID-19 to autoimmune and inflammatory sequelae (123). Especially, the exacerbated, uncontrolled production of proinflammatory cytokines (cytokine storm) has a tremendous impact on the activation of immune cells, such as neutrophils, in general and on the bystander activation of potentially autoreactive B and T cells in particular (123).

## The molecular mimicry concept as a basis to model autoimmunity

Due to its putative critical role in the development of autoimmune disease, molecular mimicry has been widely used in animal models. In the majority of such models, mice have been either directly infected with pathogens or challenged with antigens in combination with adjuvants. Often the mimicry host counterpart is provided through a tissue-specific expression of a transgene. The advantage of such models, over spontaneous models for autoimmune diseases, lays in the use of well-characterized antigens and the ability to define the start of the pathogenesis. To induce an actual autoimmune disease in these models, a sufficiently high number of autoreactive lymphocytes with adequate affinity for the target autoantigen must be generated to inflict substantial damage on the target tissue. Consequently, many models face the challenge of strong immune tolerance, particularly when the target antigen is also expressed in the thymus or when it is located within an immune-privileged site such as the eye or liver. The lymphocytic choriomeningitis virus (LCMV) has been used as something like the favorite pet by immunologists over decades and led to several breakthroughs in immunological research, such as the discovery of MHC-restriction by Doherty and Zinkernagel (126). Thus, in transgenic models the glycoprotein (GP) and nucleoprotein (NP) of LCMV, have been widely used as well-defined target autoantigens. For example, these model antigens have been expressed specifically in the β-cells of the pancreatic islets of Langerhans under the control of the rat insulin promoter (RIP) (127, 128). Upon infection with LCMV such transgenic RIP-LCMV mice develop T1D. Interestingly, in these transgenic mice LCMV-NP is also expressed in the thymus, whereas the expression of LCMV-GP is restricted to the pancreas (129). Thus, in RIP-LCMV-NP mice high-affinity NP-specific T cells are eliminated through central tolerance mechanisms and, as a result, T1D ensues in a delayed and milder form than in RIP-LCMV-GP mice [reviewed in (130)]. A similar mechanism operates in mice expressing an LCMV protein under the control of the MBP promoter in oligodendrocytes. In these animals, LCMV infection directs the autoimmune response toward myelin sheaths, resulting in a central nervous system disease that resembles human MS (131). In addition, transgenic mice with an albumin promotor-controlled expression of the immunodominant LCMV-GP CD8 T cell epitope in hepatocytes have been generated. Although these mice are relatively resistant to disease development, LCMV infection combined with the adoptive transfer of TcR-transgenic, GP peptide-specific T cells induce a transient AIH-like disease (132).

In all these LCMV-models for autoimmune disease an environmental trigger (LCMV) was used that carried components (GP or NP) that were identical to the target autoantigen (GP or NP). On the one hand, infection with a pathogen carrying a component identical to a target tissue antigen may provide a “perfect fit” and consequently generate a higher frequency of lymphocytes with sufficient avidity to damage the target tissue. However, on the other hand, if expressed in the thymus identical triggers might encounter strong central tolerance preventing a tolerance breakdown. Even in the absence of thymic expression, peripheral tolerance mechanisms may induce a degree of unresponsiveness to the triggering pathogen and thus limit the development of a vigorous pathogenic immune response. In contrast, infection with pathogens expressing structures that are similar, but not identical, to host antigens may circumvent pre-existing tolerance. However, autoimmunity initiated through molecular mimicry rather than molecular identity might not generate enough autoreactive lymphocytes with sufficient avidity to surpass the threshold required for clinical autoimmune disease.

Nevertheless, in such scenarios molecular mimicry might be sufficient to accelerate disease progression. In the RIP-LCMV model of T1D, an infection of RIP-LCMV-NP mice with Pichinde virus (PV), which shares a subdominant cross-reactive nucleoprotein epitope (PV-NP) with LCMV-NP, elicits only a weak anti-NP CD8 T cell response and fails to induce clinical T1D. However, when RIP-LCNV-NP mice, which normally show a slow-onset T1D, are first primed with LCMV and then infected with PV the onset of T1D is significantly accelerated (133). The reason for this acceleration is an expansion of autoreactive CD8 T cells recognizing the subdominant PV/LCMV-NP mimicry epitope resulting in a higher total number of LCMV-NP-specific CD8 T cells (133). Thus, depending on the magnitude of the cross-reactive immune response and the host’s cumulative pathogen exposure, molecular mimicry may also contribute to the acceleration and progression of autoimmunity.

A situation in which both mimicry and identity are similarly successful in inducing disease has been observed in the Theiler’s murine encephalomyelitis virus (TMEV) model for human CNS autoimmunity, like MS. A recombinant TMEV was generated that expresses either the encephalitogenic myelin proteolipid protein (PLP) T cell epitope PLP139–151 (PLP-TMEV) or a bacterial mimic peptide that is naturally expressed by *Haemophilus influenzae* (*H. influenzae*) (HI-TMEV) (134). In this model, infection with both PLP-TMEV and HI-TMEV induced MS-like CNS autoimmunity indicating that self-tolerance can be broken by both molecular identity and molecular mimicry, dependent on the degree of degeneracy in the T cell repertoire (134).

A setting in which molecular mimicry, rather than molecular identity, efficiently breaks tolerance to a natural autoantigen has been observed in the CYP2D6 mouse model of AIH (37). CYP2D6 is one of the major autoantigens in AIH type 2 and is recognized by LKM-1 antibodies in the majority of AIH-2 patients. The CYP2D6 mouse model uses wild-type mice (strains FVB or C57BL/6) expressing only murine Cyp homologues, which share substantial structural similarity with human CYP2D6 but are not identical. Infection of such mice with an adenoviral vector expressing human CYP2D6 (Ad-2D6) breaks tolerance to the endogenous murine Cyps and triggers a robust autoimmune response, resulting in chronic hepatitis characterized by extensive cellular infiltration and subcapsular fibrosis (37). Importantly, LKM-1-like specific autoantibodies are generated that exhibit the same epitope specificity as in patients with AIH and recognize both human CYP2D6 and the mouse Cyp homologues. Furthermore, human CYP2D6/mouse Cyp homologue cross-reactive CD8 and CD4 T cells are induced and migrate to the liver (37, 38). By contrast, Ad-2D6 infection of transgenic mice that express both the murine Cyps and human CYP2D6 in the liver fails to overcome T cell tolerance to human CYP2D6. These transgenic mice show almost no CD8 and CD4 T cell response to either human CYP2D6 or the mouse Cyp homologues but due to the generation of anti-CYP2D6 antibodies still develop AIH-like disease, albeit with much lower antibody titer, less hepatocellular damage, and an overall delayed onset and attenuated severity (37, 38). These findings indicate that perfect molecular identity between pathogen-derived and host-derived structures is less effective in breaking T cell tolerance than a pathogen–host pairing characterized by partial similarity. Notably, the CYP2D6-specific T cell response is directed toward regions of the human CYP2D6 molecule that display intermediate sequence similarity to the murine Cyp homologues. In contrast, no reactivity is observed against regions exhibiting either low homology, which prevents cross-reactivity, or very high homology or identity, which maintains tolerance (38). Thus, in the CYP2D6 model, the greatest potential for breaking immune tolerance appears to reside within a molecular mimicry “sweet spot” defined by intermediate sequence homology (**Figure 1**).

Generally, the expression of the target autoantigen in the thymus seems to be crucial. There are three possible scenarios of how central tolerance may influence autoimmunity triggered by molecular mimicry: First, molecular identity would provide a maximal T cell receptor cross-reactivity and no requirement for receptor degeneracy. However, T cells with high-affinity TcR might undergo negative selection if the autoantigen is expressed in the thymus. Second, optimal molecular mimicry might result in activation of pathogen-specific T cells that escaped negative selection and would also allow, within the range of TcR degeneracy, for an activation by similar autoantigens. A structural basis for such a scenario has been demonstrated for a self-epitope of the mouse dopamine beta-mono-oxygenase and the immunodominant LCMV epitope GP33. Although these mimicry partners share only 44% sequence homology they are presented in an almost identical manner by the mouse class I MHC H-2D^b^ with very similar shape, topology, and charge distribution allowing for a similar CD8 T cell activation (135). The third and possibly also the most frequent scenario, would be weak molecular mimicry in which pathogen and host share some sequence homology. Pathogen-specific T cells would not be negatively selected in the thymus, but no cross-reactive T cells would be generated due to insufficient binding to the MHC or lack of a necessary TcR degeneracy.

## Final considerations

Since the initial recognition of molecular mimicry as a defense strategy used by pathogens to escape host immune surveillance, molecular mimicry has been accepted as a mechanism by which pathogens, or in some cases xenobiotics, may be involved in the pathogenesis of many autoimmune diseases. As outlined in **Table 3**, the concept of molecular mimicry was first introduced in the 1960s as a strategy for pathogens to evade immune recognition. Through this process, the co-evolution of pathogen and host created a major challenge for the host: namely, the risk of autoimmunity arising from an aggressive immune response against pathogens that employ molecular mimicry as a defense mechanism. Indeed, beginning in the 1970s, an increasing number of associations between pathogen infections and autoimmune diseases were documented, and pathogens came to be regarded as major environmental triggers of the breakdown of self-tolerance and the development of autoimmunity. During the 1980s and 1990s, advances in experimental technologies enabled the screening of patients’ sera and isolated lymphocytes for cross-reactive antibodies and T cells, thereby contributing to the recognition of molecular mimicry as a well-defined immunological mechanism. Further advances in the generation of transgenic mice during the 1990s and 2000s made it possible to validate many presumed cases of molecular mimicry as inducers or accelerators of autoimmune disease in well-characterized animal models. In addition, it facilitated the generation of mice expressing defined autoantigens, allowing researchers to further investigate the molecular and cellular mechanisms underlying the breakdown of immune tolerance and the development of autoimmunity.

**Table 3:** Timeline of molecular mimicry as a concept.

Stage	Main concept	Gained knowledge
1960s	Pathogens may resemble host molecules to evade immune recognition	Mimicry as an evolutionary hypothesis
1970s-1980s	Pathogen infections are linked epidemiologically to autoimmune diseases	Pathogens as environmental triggers of the disease
1980s-1990s	Cross-reactive antibodies and T cells are identified	Molecular mimicry is recognized as a mechanism
1990s-2000s	Animal models demonstrate priming or amplification of autoimmune cross-reactivity and disease	Molecular mimicry can initiate and/or accelerate disease
2010s-present	Epitope similarity is integrated with genetic predisposition, tolerance, and immune regulation	Molecular mimicry is one component of a multi-factorial and multi-hit autoimmune model

Many observations and models have shaped the concept of molecular mimicry over time. In particular, advancements in animal models with the use of transgenic mice allowed a verification of early observations and predictions.

Today, the understanding of molecular mimicry is considerably more nuanced than in its early years. Early observations relied primarily on amino acid sequence similarities and a limited number of reports describing cross-reactive antibodies, which suggested that molecular mimicry might contribute to the immunopathogenesis of certain autoimmune diseases (**Table 4**). At that time, molecular mimicry was considered a relatively rare phenomenon in which a single pathogen could trigger a breakdown of self-tolerance. Owing to the lack of large databases and modern technologies, epidemiological and experimental evidence was limited, and many findings could not be reproduced in animal models. Furthermore, the identification of sequence similarity alone provided little predictive or diagnostic value (**Table 4**). The current view regards molecular mimicry as one of several mechanisms that contribute to the development of autoimmunity. Importantly, mimicry is no longer restricted to sequence similarity but also encompasses structural (conformational) and functional similarities. It is now recognized as a widespread phenomenon that contributes to the pathogenicity of many autoimmune diseases, with a few notable examples, such as GBS and ARF, for which compelling evidence supports a causal role for molecular mimicry in disease induction. In addition, substantial evidence now indicates that molecular mimicry associated with EBV acts as an accelerator of MS. Many previously proposed cases have since been reproduced in animal models, leading to broad acceptance of the molecular mimicry concept. However, with the integration of computational and experimental systems biology approaches, it has become clear that, with only a few exceptions, molecular mimicry arising from a single infectious event represents just one factor contributing to the development of autoimmunity in susceptible individuals. Consequently, emerging predictive networks that incorporate HLA binding and the identification of putative T cell mimicry epitopes are expected to improve the predictive power of risk assessment at the individual-patient level.

**Table 4:** Molecular mimicry today.

Topic	Early observations	Modern concept
Similarity	• Linear sequence similarity • Cross-reactive antibodies only	• Multi-dimensional similarity: • Linear sequence • Conformational structure • Functional groups • Cross-reactive B and T cells
Occurrence	• Pathogen defense mechanism • Rare occurrence in autoimmunity • Anecdotal evidence	• Evolutionary exploited mechanism • Widespread phenomenon • Hard evidence for some autoimmune diseases
Etiology	• Single trigger hypothesis • Pathogen infection	• Molecular mimicry as one component of several events • Part of a multifactorial autoimmune network
Evidence	• Limited experimental evidence • Weak epidemiologic datasets • Lack of reproduction in model systems	• Integrated computational and experimental systems biology • Robust reproduction in model systems
Diagnostic	• Weak predictive power	• Emerging predictive programs • Integration of HLA-fitting T cell epitopes

Whereas early observations of rare occurrences provided only weak experimental evidence and predictive power, today molecular mimicry is considered as one of many pathogenic factors involved in the etiology of autoimmune diseases.

Further, molecular mimicry might also be harnessed for therapeutic applications. Some microbial-derived antigens have been found to mimic tumor antigens (136) and recent studies have provided extensive conformational and immunological evidence for a large pool of microbial-associated antigens (MAAs) mimicking tumor-associated antigens (TAAs) (137). Indeed, T cells cross-reactive with homologous MAAs and TAAs have been identified in both healthy individuals and cancer patients (138). Thus, using MMAs exhibiting molecular mimicry to TAAs may represent a novel anticancer vaccination strategy. To date, four clinical phase I/II trials have been initiated to assess the safety and preliminary efficacy of such vaccines (see the review by Rossjohn et al. for details (138)). Across these trials, no treatment-related severe adverse events have been reported and an expansion of CD8+ T cells cross-reactive with MAAs and their corresponding mimicked TAAs has been observed in responding patients. Moreover, initial signs of clinical efficacy have been observed in all four trials (138). Hopefully, insights gained from these trials will provide a foundation for the broader application of molecular mimicry as a therapeutic strategy in cancer treatment.

## Glossary

2-OA: 2-octoynoic acid
AAPA: anti-acetylated protein antibodies
ACPA: anti-citrullinated protein antibodies
Ad-2D6: adenoviral vector expressing human CYP2D6
AIH: autoimmune hepatitis
ALD: autoimmune liver diseases
AMA: anti-mitochondrial antibodies
AMPA: anti-modified protein antibodies
ANA: anti-nuclear antibodies
ARF: acute rheumatic fever
BCR: B cell receptor
BD: Behcet’s disease
BSA: bovine serum albumin
CarP: carbamylated protein
CD: celiac disease
CHL1: neural cell adhesion molecule L1-like protein
CRYAB: alpha-crystallin B antibody
CVB4: coxsackie virus B4
CYP2D6: cytochrome P450 2D6
DAAM3: dishevelled-associated activator of morphogenesis 2
EAE: experimental allergic encephalomyelitis
EBNA-1: Epstein–Barr virus nuclear antigen 1
EBV: Epstein-Barr Virus
EMILIN3: elastin microfibril interfacer 3
αENaC: epithelial sodium channel
EV: enterovirus
FHOD3: FH1/FH2 domain-containing protein 3
FSTL1: follistatin related protein 1
FTCD: formiminotransferase cyclodeaminase
GAD65: glutamic acid decarboxylase 65
GBS: Guillain–Barré
GlialCAM: glial cell adhesion molecule
GP: glycoprotein
HAVR2/TIM3: T cell immunoglobulin and mucin-domain 3
HBV: hepatitis B virus
HCMV: human cytomegalovirus
HCV: hepatitis C virus
HIV: human immunodeficiency virus
HSP60: heat shock protein 60
HSV-1: herpes simplex virus 1
IA-2: tyrosine phosphatase insulinoma-associated antigen 2
IAC: immunoglobulin G4-associated cholangitis
IgG4: immunoglobulin G4
IVIG: Intravenous immunoglobulin
LAMP1: lysosome associated membrane protein 1
LCMV: lymphocytic choriomeningitis virus
LKM1: type 1 liver-kidney microsomal antibodies
LOS: lipooligosaccharides
MAA: microbial-associated antigen
MBP: myelin basic protein
MDBP: major DNA-binding protein
MPO: myeloperoxidase
MS: Multiple sclerosis
MYO18A: unconventional myosin-XVIIIa
NET: neutrophil extracellular traps
NE: neutrophil elastase
NP: nucleoprotein
p2C: protein 2C
pANCA: perinuclear anti-neutrophil cytoplasmic antibodies
PBC: primary biliary cholangitis
PBMC: peripheral blood mononuclear cells
PDC-E2: E2-subunit of the pyruvate dehydrogenase complex
PLP: myelin proteolipid protein
PR3: proteinase-3
PSC: primary sclerosing cholangitis
PV: Pichinde virus
RA: rheumatoid arthritis
RIP: rat insulin promoter
RNP: ribonucleoprotein
ROS: reactive oxygen species
RV: rotavirus
SARS-CoV2: Severe acute respiratory syndrome coronavirus 2
SLE: systemic lupus erythematosus
T1D: type 1 diabetes
TAA: tumor-associated antigen
TBB5: β-tubulin isotype 5
TCR: T cell receptor
TFA-protein: trifluoroacetylated-protein
TG2: transglutaminase 2
TMEV: Theiler’s murine encephalomyelitis virus
UTI: urinary tract infection
VCA: viral capsid antigen
VP1: viral protein 1
VP7: viral protein 7.
